# *In vitro* comparison of passive and continuous ultrasonic irrigation in curved root canals

**DOI:** 10.4317/jced.53023

**Published:** 2016-10-01

**Authors:** Pablo Castelo-Baz, Purificación Varela-Patiño, Giuseppe Cantatore, Ana Domínguez-Perez, Manuel Ruíz-Piñón, Ramón Miguéns-Vila, Benjamín Martín-Biedma

**Affiliations:** 1 DDS, PhD, University of Santiago de Compostela, Spain; 2 PhD, University of Santiago de Compostela, Spain; 3 PhD, University Vita-Salute San Raffaele of Milan, Italy; 4 DDS, University of Santiago de Compostela, Spain

## Abstract

**Background:**

The efficacy of endodontic irrigation procedures can be compromised by the complexity of the root canal system. Delivering irrigants to the apical third of curved canals presents a particular challenge to endodontists. This study compared the effects of two ultrasonic irrigation techniques on the penetration of sodium hypochlorite into the main canal and simulated lateral canals of curved roots in extracted teeth.

**Material and Methods:**

Two sets of simulated lateral canals were created at 2, 4, and 6 mm from the working length in 60 single-rooted teeth (6 canals/tooth, n = 360 canals). The teeth were randomly divided into three experimental irrigation groups: group 1 (n = 20), positive pressure irrigation (PPI); group 2 (n = 20), passive ultrasonic irrigation (PUI); and group 3 (n = 20), continuous ultrasonic irrigation (CUI). To assess the irrigation solution penetration, 20% Chinese ink (Sanford Rotring GmbH, Hamburg, Germany) was added to a 5% sodium hypochlorite solution and delivered into the curved root canals. The penetration of contrast solution into the simulated lateral canals was scored by counting the number of lateral canals (0-2) penetrated to at least 50% of the total length.

**Results:**

The CUI group showed significantly higher (*P* < 0.05) irrigant penetration into the lateral canals and into the apical third of the main canals. The PPI group showed significantly lower sodium hypochlorite penetration (*P* < 0.001) into the main and lateral canals compared with that in the CUI and PUI groups. Significantly higher irrigant penetration was observed in the PUI group than the PPI group.

**Conclusions:**

Using CUI as the final rinse significantly increased the penetration of irrigant solution into the simulated lateral canals and apical third of curved roots.

** Key words:**Continuous ultrasonic irrigation, curved root canals, passive ultrasonic irrigation, positive pressure irrigation, root canal irrigation.

## Introduction

One of the most important objectives of root canal therapy is to eliminate all vital and necrotic tissue, microorganisms and products of microbial degradation from the root canal system to prevent apical periodontitis (1). Root canal irrigation with an antimicrobial solution is considered an essential part of chemical/mechanical canal preparation (2). The complexity of the root canal system complicates the shaping and cleaning procedures performed by hand or using rotary instrumentation techniques (3-5). Isthmi, fins, webs, anastomoses and other irregularities within the root canal can hinder the irrigant from dissolving the organic tissues within the canal and destroying the bacterial biofilm (6).

The apical third of the root canal seems to be the most difficult part to clean because of its complex anatomy and relatively small diameter (7), which can impede the effective flow of the irrigant (8). Furthermore, root canals often have a curvature (9), which can reduce the cleaning efficacy of several irrigation techniques (10,11). Positive pressure irrigation (PPI) produces irrigant exchange no farther than 1.0 mm beyond the needle tip (12) and is ineffective in flushing debris from the apical third of the canal without adjunctive agitation methods (13). Agitation techniques improve the apical cleaning efficacy, mechanically and chemically, by enhancing the irrigation dynamics (4).

The synergistic effects of ultrasonic energy and sodium hypochlorite in aqueous solution appear to be especially effectual (14). Currently, passive ultrasonic irrigation (PUI) is probably the most established method to activate sodium hypochlorite after root canal instrumentation (4,15,16). PUI is employed to activate an irrigant using an ultrasonically activated file or tip that is not used for canal preparation. The PUI procedure encompasses the placement of the irrigant using a syringe and the subsequent activation and delivery of the irrigant through the ultrasonic hand piece. Recent *in vitro* studies have confirmed the efficacy of PUI in removing pulp tissue from lateral canals (15,17) in straight canals and curved canals (18,19). However, complete penetration of the irrigant cannot be achieved in all phases of root canal treatment (20).

Gutarts *et al.* (3) proposed the use of an ultrasonically activated needle placed in the canal through which the sodium hypochlorite would flow, enabling the irrigant to be replaced continually (continuous ultrasonic irrigation, CUI). *In vivo* and *in vitro* studies demonstrated the efficacy of this method in cleansing inaccessible zones of the endodontic system (17,21). However, the efficacy of the CUI technique in curved canals is unknown. Likewise, it is unclear how far the CUI can reach and the effect of the root canal curvature on the depth of irrigant penetration. Therefore, the aim of this study was to compare the effects of three irrigation systems (CUI, PUI and PPI) on irrigant delivery into the apical third and simulated lateral canals of curved roots in cleared extracted teeth.

## Material and Methods

-Tooth preparation

After the approval of the ethical committee (Comité de Ética de Santiago-Lugo; 2016/269), 60 extracted human single-rooted teeth with fully formed apices of maxillary lateral incisors that had not undergone prior endodontic treatment were selected. All samples had root curvatures of 20-30º as determined by the method of Schneider (22), and the curvature started in the last 5 mm in all specimens. Teeth included in this study had a radius between 3 and 5mm. Each tooth was used in one experiment only. After debriding the root surface, specimens were immersed in a 5.25% NaOCl solution for 1 h and then stored in saline until preparation. The presence of a single canal was verified radiographically and by direct exploration. The same operator performed all experimental procedures. Each specimen was sectioned to obtain a working length of 16 mm, as described previously (17). The working length was established under a microscope (M525 F40; Leica, Heerbrugg, Switzerland) at 10× magnification with the tip of the instrument visible at the apical foramen. Each root canal was preflared using K-Flexofiles (Dentsply Maillefer, Ballaigues, Switzerland) up to #20 and then shaped using GTX 20.04, 20.06, 30.06 (Dentsply Maillefer). Irrigation was performed with a 30-G needle (ProRinse; Dentsply Tulsa Dental Specialties, Tulsa, OK) using 3-mL 5.25% NaOCl after each filing. The irrigation needles were introduced passively up to 2 mm from the working length, and the rate of delivery was fixed at 3 mL/min. The total irrigation time was 10 min/specimen. After instrumentation, all teeth were rinsed for 3 min with 3-mL 10% EDTA (Tubuliclean; OGNA) followed by a 3-min final rinse with 5.25% NaOCl. After drying with paper points, the roots were inspected under the microscope at 10× magnification to verify the canal cleanliness and absence of cracks.

After completing the shaping procedures, the teeth were cleared using the modified technique described by Robertson and Leeb (23) and prepared as described by de Gregorio *et al.* (5) Briefly, teeth were submerged in 5% nitric acid for 36 h, and the solution was renewed every 8 h. Once decalcified, all samples were cleared with tap water for 3 min, and lateral canals were created by inserting a 06 C+ file (Dentsply Maillefer) from the buccal to the lingual wall at 2, 4, and 6 mm from the working length perpendicular to the external surface. Samples were dehydrated in ascending grades of ethyl alcohol and submerged in 99.9% methyl salicylate for clearing and rehardening of dental tissues as described by Gregorio *et al.* (5) A total of 360 simulated lateral canals were created (6 canals/tooth, 2 lateral canals at each level).

To simulate the clinical situation, a closed system was created by coating each root with soft modeling wax (Cera Reus SA, Reus, Spain), and this coating sealed the apical foramen and lateral canals at all three levels (modeling wax only touch the walls, it can´t penetrate into lateral canals). During this procedure, a GTX 40.06 gutta-percha point (Dentsply Maillefer) was introduced into the canal to the working length to prevent wax penetration into the canal space.

-Contrast Solution

A contrast solution containing 5% NaOCl (80%) and 20% Chinese ink (Sanford Rotring GmbH, Hamburg, Germany) was prepared and delivered to the prepared root canals.

-Experimental Groups

The teeth were randomly divided into three experimental irrigation groups: group 1 (n = 20), positive pressure irrigation (PPI); group 2 (n = 20), passive ultrasonic irrigation (PUI); and group 3 (n = 20), continuous ultrasonic irrigation (CUI). Irrigation was performed in the PPI (control) and PUI groups with slight modification of the methods described by de Gregorio *et al.* (5). Irrigation was performed in the CUI group with slight modification to the manufacturer’s instructions. The irrigation time was identical for the three groups (1 min). All procedures were recorded under a dental operating microscope.

The teeth in group 1 (n = 20, control) were irrigated with PPI (1 min) using a 30-G ProRinse needle and a syringe at 2 mm from the working length. A total volume of 6 mL of contrast solution was delivered. The solution was not dynamically activated in this group.

The teeth in group 2 (n = 20) were irrigated using PUI. Contrast solution (a total volume of 2 mL) was delivered into the teeth using a 30-G ProRinse needle, and the solution was left in the root canals. Ultrasonic activation was performed with a blank ESI file (EMS, Nyon, Switzerland). The file was inserted passively to 1 mm from the working length and activated during 20 s using a power setting of 6, as recommended by the manufacturer. The procedure was repeated three times, with a total volume of 6 mL of contrast solution and a total activation time of 1 min for each tooth.

The teeth in group 3 (n = 20) were irrigated using CUI. Active ultrasonic activation was performed using ProUltra PiezoFlow needles mounted on a Suprasson P5 Booster ultrasonic unit. A 10-mL syringe containing contrast solution was attached to the luer-lock connection on the UI needle. The length at which the needle began to bind against the canal walls was determined, and the needle was then pulled back ~1 mm and the silicon stopper was set to this depth. The insertion depth did not exceed 75% of the working length established for instrumentation; it was placed before the curvature. The inactive needle was placed into the canal, and the solution was delivered. Once irrigant filled the canal, the ultrasonic unit was activated with the power set to level 6. Maintaining a continuous irrigation flow of 6 mL/min, the active ultrasonic tip was gently moved up and down in a consistent straight-line path from the canal entrance to the stopper setting. The total activation time was 1 min, and a total volume of 6 mL of contrast solution was delivered.

-Evaluation Criteria

The samples were evaluated by direct observation of images recorded under the dental operating microscope using the criteria described by de Gregorio *et al.* (5) The orientation of all samples was standardized in relation to the recording microscope to produce similar images for all groups.

The penetration of contrast solution into the simulated lateral canals was scored by counting the number of lateral canals (0-2) penetrated to at least 50% of the total length. The outcome was assessed in each tooth at each of the three working lengths (2, 4 and 6 mm). Each sample scored by one trained evaluator who was blinded to the group assignment.

-Statistical Analysis

The mixed models test was used to analyze and compare irrigant penetration to the working length and into the lateral canals. *P*-values <0.05 were considered to indicate statistical significance.

## Results

**Figure 1:**
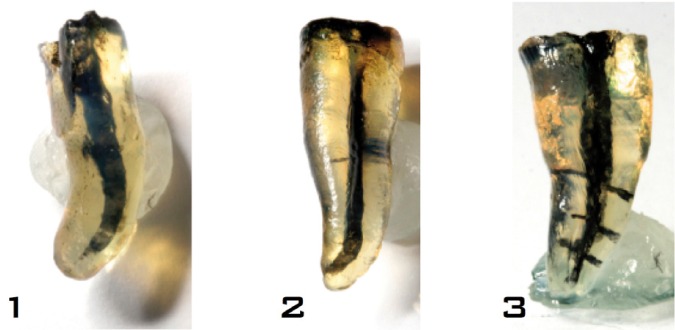
1.-PPI. 2.- PUI. 3.- CUI.

The flow of irrigant into the lateral canals and the working lengths of the root canals were analyzed in all samples (n = 20) in each group. A representative sample from each group is shown in figure 1. The contrast solution did not reach the working length in the group 1 samples (0%). However, the contrast solution reached 40% of the working length in group 2 (PUI) and 90% of the working length in group 3 (CUI). Penetration in group 1 differed significantly from that in the other two groups (*P* < 0.001). Furthermore, penetration in group 2 differed significantly from that in group 3 (*P* < 0.001).

Overall penetration into the lateral canals was 0% in group 1 (PPI), 46% in group 2 (PUI), and 92% in group 3 (CUI,  Table 1). These values differed significantly among the groups (*P* < 0.036). These results were confirmed in separate analyses of the three levels (2, 4 and 6 mm), which indicated a lower penetration rate in group 1 than in groups 2 and 3.

**Table 1:**
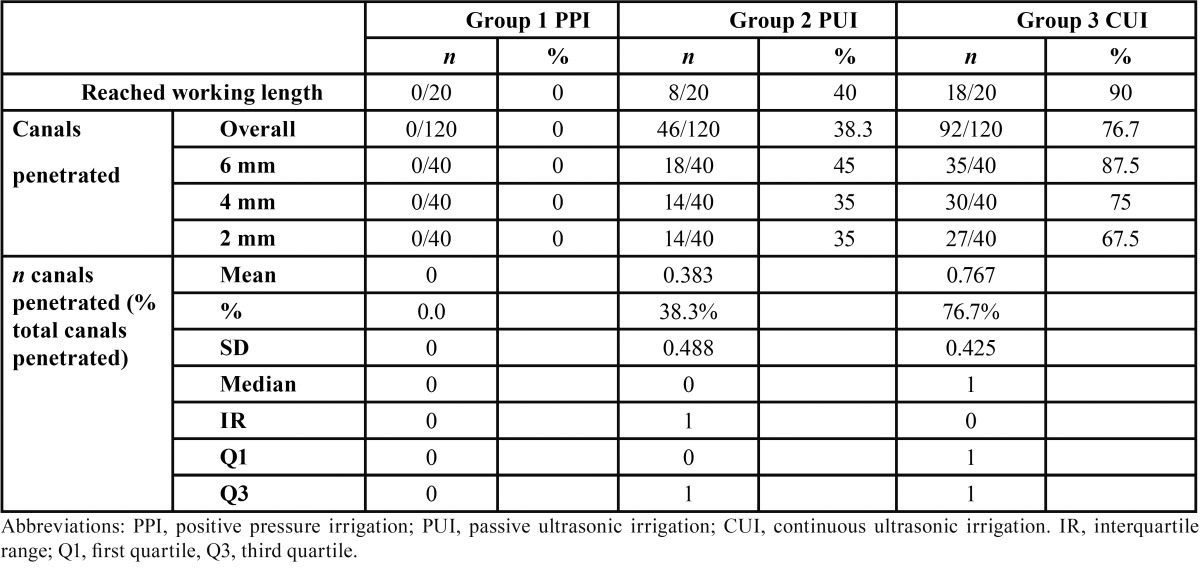
Comparison of three irrigation systems: working length penetration and lateral canal penetration of the irrigant.

## Discussion

Several root canal irrigation techniques and systems have been reported to improve final irrigation before obturation (24). Irrigant activation in the root canal system can enhance irrigant dispersal (25) and improve mechanical cleaning of the root canal by effective fluid flow dynamics (26). However, to our knowledge, no studies have investigated CUI into curved canals, and few studies have reported on PUI in curved canals or roots. The aim of this study was to compare the effectiveness of three irrigation techniques (PPI, PUI and CUI) in terms of delivering irrigant to the working length and into the lateral canals in curved canals.

In this *in vitro* investigation, we compared the efficacy of two ultrasonic techniques (PUI, CUI) with one traditional (PPI). To simulate the clinical situation, we used an *in vitro* closed-end canal design that closely replicates the *in vivo* scenario (27) in which the apical foramen is enclosed by the periodontal tissues (3,22). This design forces the irrigants to exit the canal coronally rather than apically or laterally (5). As shown in our previous study (17) and in a study by de Gregorio *et al.* in a similar model (5), the PPI technique does not deliver irrigant to the working length and does not generate enough pressure to force the irrigants within the simulated lateral canals. Tay *et al.* suggested that the inability of PPI to reach the apical third may be caused by the presence of an apical vapor lock (28) created by the organic decomposition of NaOCl into a bubble of carbon dioxide and ammonium (5) that adversely affects the debridement efficacy of PPI in a close-end canal design. We compared the two PUI and CUI ultrasonic techniques in curved canals. Although we employed the new nickel-titanium ESI file (EMS, Nyon, Switzerland)—which is more flexible than the stainless steel ultrasonic tips used in a previous study (17) —we found that PUI delivered adequate irrigant penetration into the apical third of only 40% of the samples. This finding suggests that PUI does not provide sufficient force to overcome the apical vapor lock after a curvature. In contrast, CUI delivered favorable penetration of sodium hypochlorite up to the working length in 90% of the samples. This group showed enough force to overcome the vapor lock, and a statistically significant difference was observed between the two groups (*P* < 0.001). This finding might be due to the continuous exchange of solution provided by the CUI technique and the optimized activation of the solution as it passes through the ultrasonically energized needle. Both ultrasonic techniques performed significantly better than PPI, as measured by irrigant penetration into the artificially created lateral canals and into the apical third. These results confirm those obtained previously, in which the efficacy of CUI was found to be significantly higher than that of PPI (18).

The CUI group received a greater volume of irrigant within the root canal system during the endodontic treatment, which is considered to be a key factor in debris removal and disinfection (29). This technique, however, has several disadvantages because its action may push the irrigants beyond the distance at which the frontal pressure of a syringe normally operates when used for canal detersion (30). Such force might compromise the safety of the cleansing procedure because of the severe consequences if sodium hypochlorite were extruded into the periapical tissue. Other techniques, such as the negative-pressure cleansing system, appear to be safer than manual and ultrasonic irrigation with particular reference to the ultrasonic needle; however, no clear superiority of this negative-pressure system in terms of penetration and cleansing of the endodontic space has been reported (30). To prevent the possibility of creating a ledge on the canal walls when using the CUI technique, we selected roots with curvatures in the final 5 mm. Therefore, we reduced the risk of these ledges to allow adequate evaluation of the CUI technique into curved canals.

This investigation compared the two ultrasonic techniques (PUI and CUI) with PPI regarding the penetration depth in the simulated lateral canals. The CUI group exhibited contrast solution penetration in a significantly higher number of lateral canals, as was shown in straight canals in a previous study (17). This result might be explained by the continuous exchange of solution and the optimized activation of the solution as it passes through the ultrasonically energized needle. This finding confirms that CUI delivers irrigant more effectively than PPI or PUI into lateral canals in curved and straight roots.
